# An Evaluation of Renal Sinus Fat Accumulation Using the Anteroposterior Diameter of the Renal Sinus on a Computed Tomography Axial Image

**DOI:** 10.7759/cureus.58006

**Published:** 2024-04-10

**Authors:** Yasuhiro Inokuchi, Tsuneyuki Takashina, Yusuke Hayashi, Jo Sakihara, Masahiro Uematsu, Hiromasa Kurosaki

**Affiliations:** 1 Department of Radiology, Edogawa Hospital, Tokyo, JPN; 2 Department of Radiology and Radiation Oncology, Edogawa Hospital, Tokyo, JPN

**Keywords:** multidetector computed tomography, ckd (chronic kidney disease), renal sinus fat invasion, hyper blood pressure, diagnostic imaging, obesity

## Abstract

Backgrounds and objectives

Renal sinus fat (RSF) is an indicator of obesity-related complications. However, the measurement and imaging process are complicated. For a simple measurement of RSF, we focused on the kidney's shape change caused by RSF accumulation. Thus, this study aimed to investigate whether the anteroposterior diameter of the renal sinus (APDRS) on a computed tomography (CT) axial image is useful for evaluating RSF accumulation.

Materials and methods

The correlation between APDRS and RSF was investigated in 98 outpatients who underwent abdominal CT. In addition, the correlation between APDRS or RSF and obesity indicators (estimated glomerular filtration rate from serum creatinine levels (eGFRcreat), body mass index (BMI), and visceral adipose tissue (VAT)) was also investigated. We classified patients based on the presence or absence of at least one underlying disease (chronic kidney disease (CKD), cardiovascular diseases (CVD), hypertension, and type 2 diabetes (T2D)) and investigated significant differences between the two groups at APDRS and RSF. The intraclass correlation coefficient (ICC) was also calculated for APDRS.

Results

There was a strong positive correlation between RSF and APDRS (r = 0.802, P < 0.01). The obesity indicators (eGFRcreat, BMI, and VAT) were correlated with RSF and APDRS (P < 0.01). Out of 98 outpatients, 48 had at least one underlying disease. There were statistically significant differences in APDRS and RSF between the patients with and without at least one of the underlying diseases caused by obesity (P < 0.01). The inter-reader ICC for the measurement of the APDRS was 0.98.

Conclusions

APDRS on a CT axial image may be useful for the evaluation of RSF accumulation.

## Introduction

Obesity is a common condition worldwide epidemic, and its prevalence is on the rise [1]. Obesity causes a myriad of metabolic complications, including chronic kidney disease (CKD), cardiovascular disease (CVD), hypertension, type 2 diabetes (T2D), COVID-19, and an increased risk of many types of cancer [2-8]. Obesity is closely associated with ectopic fat deposition, and a special site of visceral fat deposition is the renal sinus. Renal sinus fat (RSF) accumulation is known to be correlated with obesity indicators such as the body mass index (BMI) and visceral adipose tissue (VAT) [9,10]. Furthermore, RSF accumulation has been previously confirmed in humans and animal models to affect renal structure, function, and vasculature [11-13]. Foster et al. [13] reported in the Framingham Heart Study that RSF accumulation is a common condition that predisposes people to hypertension and CKD by exerting pressure on the intrasinus renal vein. Renal vein compression leads to the activation of the renin-angiotensin-aldosterone system (RAAS), which affects renal hemodynamics and causes renal venous hypertension. This phenomenon increases the glomerular hydrostatic pressure and decreases the glomerular filtration rate (GFR) [4,11]. Therefore, RSF is considered one of the predictors of obesity-related complications. The degree of obesity can be easily evaluated by measuring the BMI [14]. However, the effects of such organ-specific fat accumulation cannot be captured by conventional anthropometric measurements of obesity; thus, imaging techniques using computed tomography (CT) and magnetic resonance imaging are used to measure the ectopic fat content of the renal sinus. However, the measurement methods reported in some previous studies [9,13,15-17] tend to require special equipment and technical skills, and the complicated and time-consuming nature of these measurement methods potentially takes a toll on their accuracy. Therefore, we thought that a simpler measurement method was needed to use RSF in clinical settings. We focused that an increase in renal sinus internal pressure due to RSF accumulation may affect the shape of the anteroposterior diameter of the renal sinus (APDRS), which is the pathway of blood vessels and ureters. Because measurement of APDRS on a CT axial image is a simple method, it is easy to follow anywhere a monitor with a measurement tool. Thus, this present study aimed to determine whether the APDRS measured on a CT axial image is useful as a simple assessment method for RSF accumulation.

## Materials and methods

Patients information

**Figure 1:**
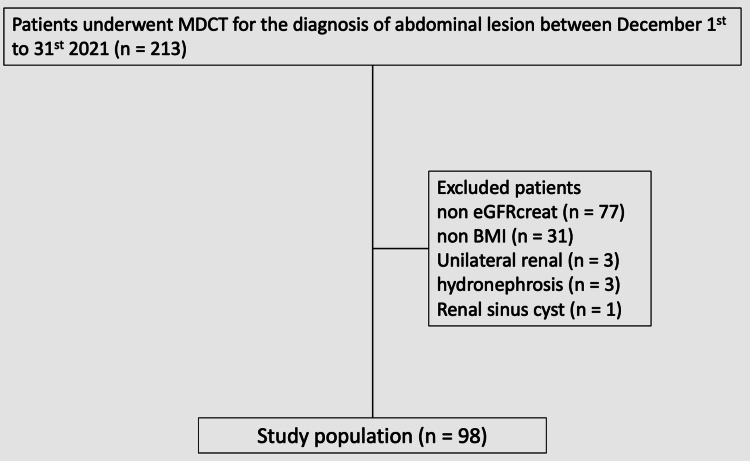
Flow chart of patient enrollment MDCT: multidetector computed tomography; eGFRcreat: estimated glomerular filtration rate from serum creatinine levels; BMI: body mass index

This study was approved by the relevant institutional review board, and the requirement for patients’ informed consent was waived due to its retrospective nature. To compare the APDRS and RSF, we collected data from 213 consecutive outpatients who underwent non-contrast-enhanced abdominal-to-pelvis CT for the diagnosis of abdominal disease (from December 1 to 31, 2021) from our institutional database. We used measurements of the indicators of obesity (eGFR from serum creatinine levels (eGFRcreat), BMI, and VAT) that were obtained within one month before the CT. We excluded 105 patients who had no eGFRcreat data (n = 77), no BMI data (n = 31), a single kidney (n = 3), hydronephrosis (n = 3), and a renal sinus cyst (n = 1) (Figure 1).

CT protocol

A 64-detector CT scanner (Revolution EVO; GE Healthcare, Chicago, IL) with a fixed tube voltage of 120 kVp and an automatic tube current modulation program was used. CT parameters were as follows: collimation, 0.625 mm; detector configuration, 64 × 0.625 mm; noise index, 13; pitch factor, 0.984:1; gantry rotation time, 0.5 s. All transverse CT images were reconstructed at 5 mm-thick sections, with the intensity of the adaptive statistical iterative reconstruction set at 50%. The CT scan was performed in the cephalocaudal direction, and all CT images were obtained from the top of the liver to the bottom of the ischium.

Measurement of APDRS

**Figure 2:**
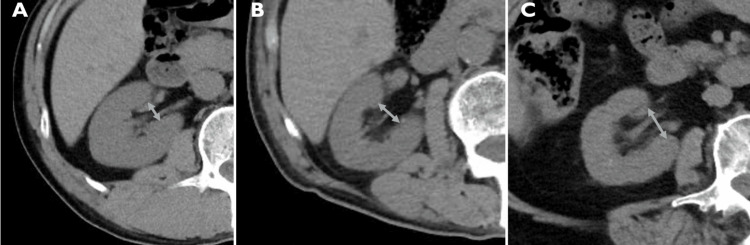
Measurement of APDRS (A) 32-years-old man, APDRS = 7.9 mm (double-headed grey arrow); (B) 74-years-old man, APDRS = 12.3 mm (double-headed grey arrow); (C) 78-years-old man, APDRS = 17.3 mm (double-headed grey arrow) APDRS: anteroposterior diameter of the renal sinus

The APDRS was defined as a line connecting the ventral side-edge and the dorsal side-edge of the inner cut-edge between the right renal sinus and the renal hilum in the axial CT image (Figure 2). The slice for measurement was selected from the center of the range of the entire renal hilum, rendered on a CT axial image. If the total range was an even number of slices, then the larger APDRS of the two center slices was selected. Measurement slice selection was conducted as well as in a previous study [17]. APDRS values of all patients were measured using a measurement tool on the picture archiving and communication system (PACS) monitor (Synapse; FUJIFILM, Tokyo, Japan) by a radiological technician with 20 years of experience in MDCT imaging.

Measurement of RSF

**Figure 3:**
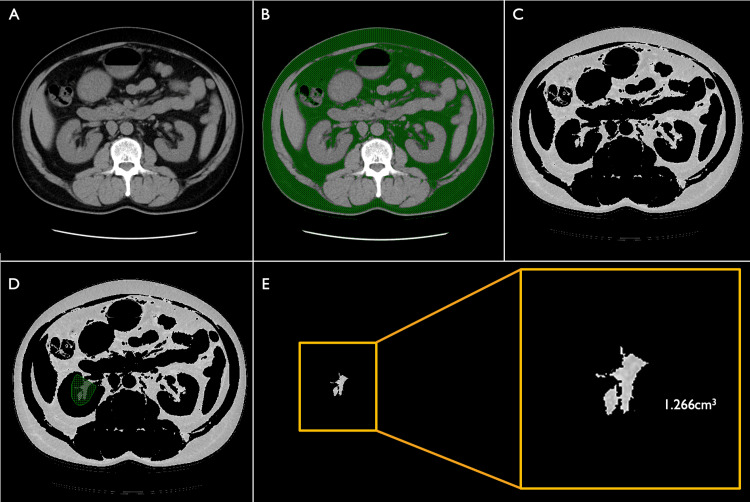
Measurement of RSF Measurement of RSF was performed according to the following procedures: 52-year-old man, RSF = 1.266 cm^3^. (A) A 5-mm-thickness slice image used for APDRS measurement was loaded into the workstation. (B) To identify the fat in the renal sinus, the CT threshold value was set from -195HU to -45HU, as described in previous studies [17]. (C) Fat-weighted images were created. (D) Next, only the fat within the right renal sinus was remaining. (E) RSF measurements were completed. APDRS: anteroposterior diameter of the renal sinus; CT: computed tomography; RSF: renal sinus fat; HU: Hounsfield units

An image with a slice thickness of 5 mm used for APDRS measurement was loaded into the workstation (Advanced Workstation Volumeshare 5; GE Healthcare, Chicago, IL). To identify the fat in the renal sinus, the CT value threshold is set from -195 Hounsfield units (HU) to -45 HU, as in previous studies [17], fat-weighted images are created. Next, only the fat within the right renal sinus is left. RSF volumetric measurements were performed (Figure 3).

Measurement of other parameters

VAT measurements were performed as they were done in previous studies [18]. The CT values threshold is set from -195 HU to -45 HU [17]. The measurement slice level was the umbilicus. VAT was performed using the same workstation, and so was the measurement of RSF. Information about each participant’s eGFRcreat, age, BMI, CKD, CVD, hypertension, and T2D were derived from data obtained within one month before MDCT imaging. The eGFRcreatwas calculated using the following equation [19]:

$$eGFRcreat [Male] = 194 &times; Cr ^{&minus;1.094} &times; age ^{&minus;0.287}$$

$$eGFRcreat [Female] = 194 &times; Cr ^{&minus;1.094} &times; age ^{&minus;0.287} &times; 0.739$$

BMI was calculated using the following equation:

$$BMI = weight (kg)/(height (m) &times; height (m))$$

Statistical analysis

To investigate whether the APDRS could be used as a simple method for the evaluation of RSF, we investigated the correlation between those using Pearson's product-moment correlation. Additionally, we investigated the correlation between RSF or APDRS and indicators of obesity (eGFRcreat, BMI, and VAT) using Pearson's product-moment correlation, respectively. We investigated significant differences between the presence and absence of at least one underlying disease caused by obesity at RSF or APDRS using the two-sample t-test. The underlying diseases group included CKD, CVD, hypertension, and T2D.

The inter-reader reproducibility of APDRS measurements was evaluated using the intraclass correlation coefficient (ICC [2,1]), for two readers (radiological technicians with 20 years and four years of experience in CT imaging). The threshold for statistical significance was set at P < 0.05. All statistical analyses were performed using EZR software (Saitama Medical Center, Jichi Medical University, Saitama, Japan).

## Results

**Figure 4:**
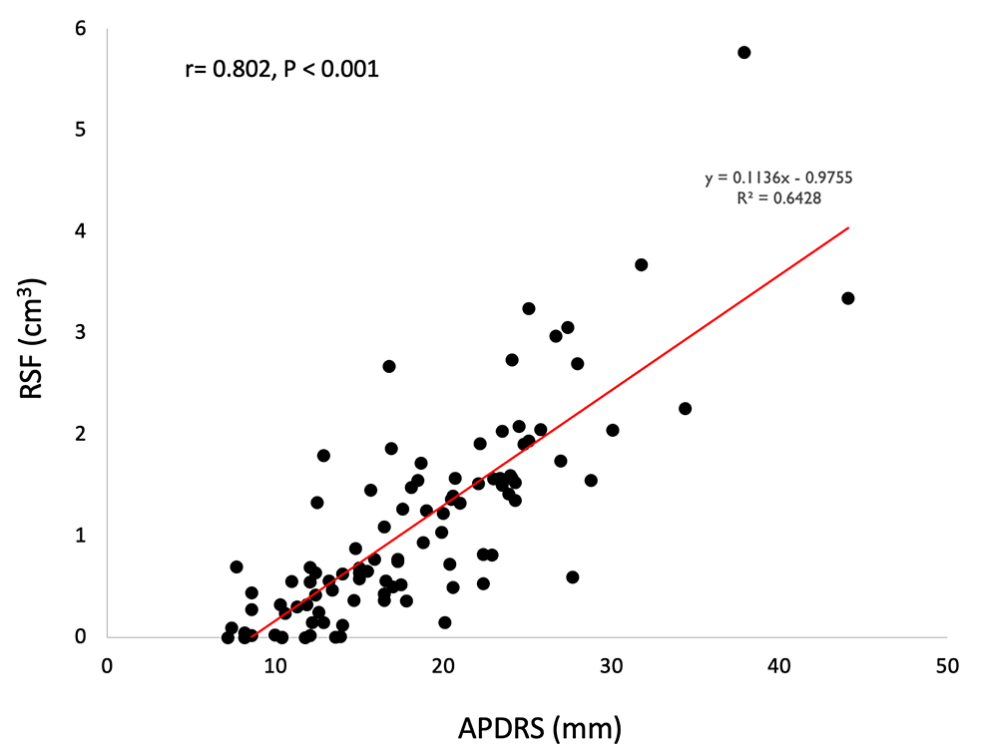
Scatter plot of the correlation between APDRS and RSF There was a strong correlation between APDRS and RSF. APDRS: anteroposterior diameter of the renal sinus; RSF: renal sinus fat

The laboratory parameters and clinical data of 98 outpatients are summarized in Table 1. Among the 98 outpatients, the mean age was 67.0 years and 64 (65.3%) were men. The mean eGFR was 66.8 mL/min/1.73m^2^. Furthermore, the mean BMI, RSF, VAT, and APDRS in the obesity indexes were 24.4 kg/m^2^, 1.1 cm^3^, 131.8 cm^2^, and 18.2 mm, respectively, and four participants had an undetectable RSF measurement. Figure 4 shows a correlation between RSF and APDRS. There was a strong positive correlation between RSF and APDRS using Pearson’s product-moment correlation (r = 0.802, P < 0.01).

**Table 1:** Patient characteristics. APDRS: anteroposterior diameter of the renal sinus; BMI: body mass index; CKD: chronic kidney disease; CVD: cardiovascular disease; eGFRcreat: estimated glomerular filtration rate from serum creatinine levels; RSF: renal sinus fat; VAT: visceral adipose tissue * Data are mean ± standard deviation (range).

Subjects	Variables
No. of patients	98
Male : Female	64 : 34
Age* (year)	67.0 ± 14.7 (24-89)
eGFRcreat* (ml/min/1.73m^2^)	66.8 ± 23.0 (3-133)
BMI* (kg/m^2^)	24.4 ± 3.8 (3-133)
RSF* (cm^3^)	1.1 ± 0.9 (0-5.7)
VAT* (cm^2^)	131.8 ± 71.1 (6.7-293.4)
APDRS* (mm)	18.2 ± 6.9 (7.2-44.1)

**Figure 5:**
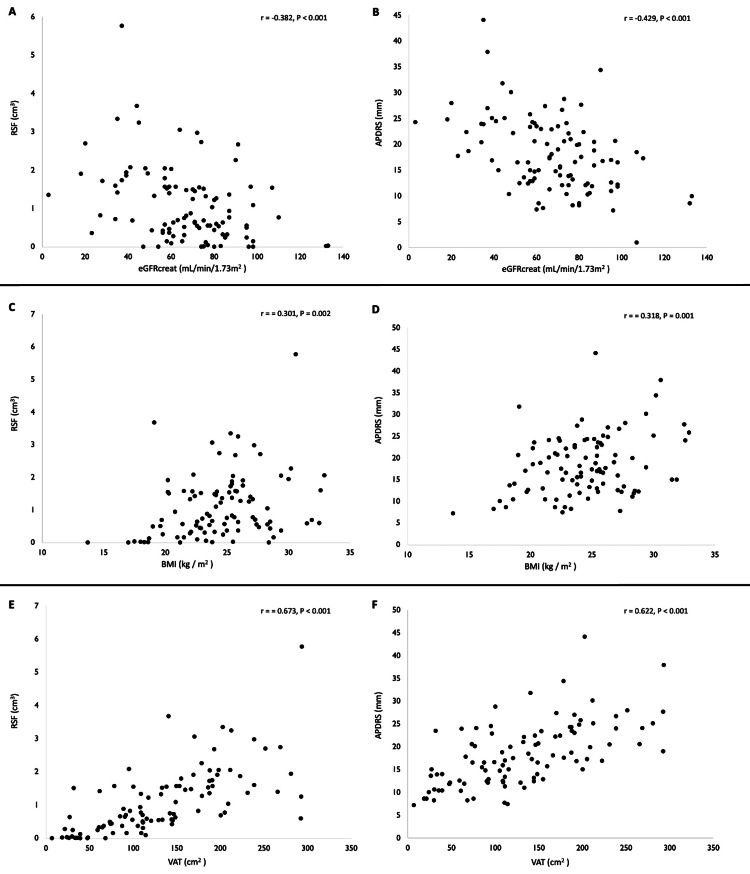
Scatter plot of the correlation between each group (A) RSF and eGFRcreat, (B) APDRS and eGFRcreat, (C) RSF and BMI, (D) APDRS and BMI, (E) RSF and VAT, (F) APDRS and VAT. Significant correlations were observed in all groups. APDRS: anteroposterior diameter of the renal sinus; BMI: body mass index; eGFRcreat: estimated glomerular filtration rate from serum creatinine levels; RSF: renal sinus fat; VAT: visceral adipose tissue

**Figure 6:**
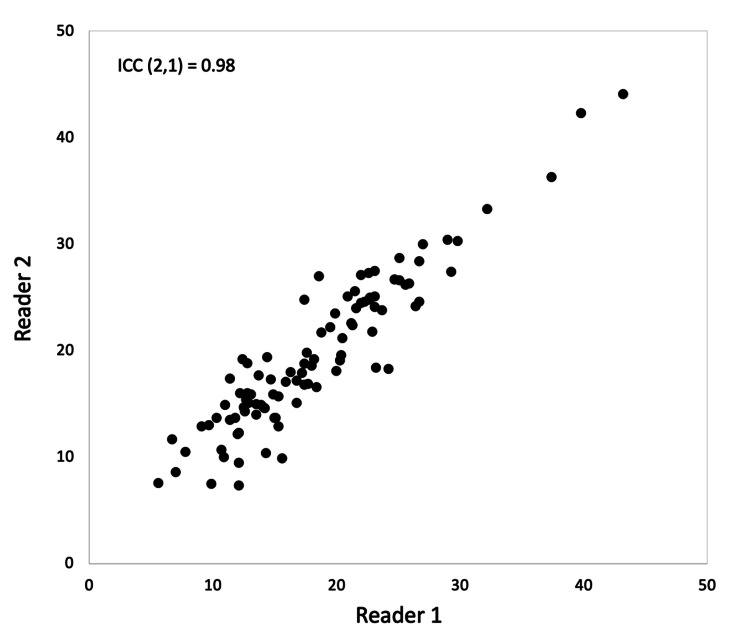
Scatter plot of intra-class correlation coefficient at APDRS measurement Reader 1: Radiological technicians with 20 years of experience in CT imaging. Reader 2: Radiological technicians with four years of experience in CT imaging. APDRS: anteroposterior diameter of the renal sinus; ICC: intraclass correlation coefficient

Figure 5 shows the correlation of RSF and APDRS with eGFRcreat, BMI, and VAT. Correlations for all combinations were observed using Pearson’s product-moment correlation (P < 0.01). APDRS showed a better correlation with eGFR and BMI than RSF, RSF, however, showed a better correlation with VAT than APDRS. Of all the three items, VAT had the largest correlation coefficient with RSF and APDRS. Out of 98 outpatients, 48 had at least one underlying disease. The numbers of underlying diseases caused by obesity were as follows: (1) 24 patients, (2) 16 patients, (3) seven patients, (4) one patient. The number of underlying diseases was highest in HT, followed by CKD, CVD, and T2D. There were statistically significant differences in APDRS and RSF between the patients with and without at least one of the underlying diseases caused by obesity. Other obesity indicators (BMI and VAT) also show significant differences in the two groups, the t and P values of BMI indicated the most inferior values of the indicators. Furthermore, measurements of RSF and APDRS in those without the underlying disease group decreased than those with at least one of the underlying diseases caused by the obesity group. Similar phenomena were also found in other obesity indicators (BMI and VAT) (Table 2). The inter-reader ICC for the measurement of the APDRS was 0.98, it showed excellent intra-reader reproducibility (Figure 6).

**Table 2:** Patient characteristics in each group APDRS: anteroposterior diameter of the renal sinus; BMI: body mass index; CKD: chronic kidney disease; CVD: cardiovascular disease; eGFRcreat: estimated glomerular filtration rate from serum creatinine levels; HT: hypertension; RSF: renal sinus fat; T2D: type 2 diabetes; VAT: visceral adipose tissue * Data are mean ± standard deviation. † The t and P values were obtained with the two-sample t-test.

Subjects	Without the underlying diseases	With at least one of the underlying diseases	t values†	P values†
No. of patients	50	48		-
Male: Female	30: 20	34: 14		-
Age (year)	61.9 ± 16.6	72.2 ± 10.2	-3.66	<0.01
eGFRcreat* (mL/min/1.73m^2^)	74.6 ± 20.6	58.7 ± 22.8	3.63	<0.01
BMI* (kg/m^2^)	23.6 ± 3.8	25.2 ± 3.3	-2.15	0.03
RSF* (cm^3^)	0.70 ± 0.60	1.51 ± 1.13	-4.39	<0.01
VAT* (cm^2^)	107.5 ± 64.5	157.1 ± 69.4	-3.65	<0.01
APDRS* (mm)	16.0 ± 5.5	20.6 ± 7.5	-3.45	<0.01
Etiology (CKD/CVD/HT/T2D)	-	(28/18/38/16)		-

## Discussion

In this study, we investigated whether APDRS is useful as a simple method for the evaluation of RSF accumulation and found a strong positive correlation between APDRS and RSF. Furthermore, APDRS had earlier shown correlations with obesity-influencing factors (eGFRcreat, BMI, and VAT), as well as RSF. The APDRS was also associated with underlying diseases caused by increased RSF, suggesting a close relationship between the APDRS and RSF. Additionally, since there was an approximate relationship between RSF and obesity-influencing factors in this study (as well as in a previous study), we considered that the data collected from this study’s sample were reliable. The relationship between APDRS and RSF is considered to be as follows. We believe that the expansion of APDRS occurs alongside the widening of the renal sinus lumen due to RSF accumulation. Montani et al. [11] reported that various renal structures can become compacted as fat deposits fill the renal sinus. The place where the pressure from the compression of the accumulated RSF can escape is the junction between the APDRS and the retroperitoneal space, and the increase in the amount of ectopic fat in the renal sinus can be judged based on the expansion of the APDRS. In addition, arteriosclerosis-induced renal atrophy may be involved in the pathogenesis of APDRS expansion [20-22]. The activation of the RAAS by RSF accumulation is known to promote atherosclerosis, leading to renal atrophy and renal fibrosis [22]. Atrophy of the kidney near the renal portals leads to APDRS enlargement. This phenomenon can be cited as a factor in which APDRS could visually demonstrate the mechanism of renal function deterioration and had a better correlation with the eGFRcreat than with the RSF. The ICC of APDRS measurement between readers (ICC = 0.98) was better than that reported in a previous study conducted on RSF measurement by Foster et al. [17], who established the usefulness of volumetric RSF using a central slice. Most previous studies [9,13,15-17] entailed complicated procedures such as RSF tracing, fat threshold selection, and fat weight imaging; however, in this study, the slice was selected according to the report of Foster et al. [17], and we only drew a line connecting the ventral side-edge to the dorsal side-edge of the inner cut-edge between the right renal sinus and the renal hilum. Therefore, we considered that the present study, which had only a few work processes, demonstrated good reproducibility. Furthermore, the RSF measurement techniques reported in those previous studies all require specialized equipment. In contrast, APDRS can be measured using only the tools built into PACS monitor and CT instruments. Therefore, APDRS measurements are considered superior in terms of reproducibility and convenience.

However, there were several limitations in the study. The study involves only 98 patients, potentially limiting the generalizability of the findings. (1) Retrospective analysis: Data collection is retrospective, potentially introducing bias. (2) Single-center study: Results may not be applicable to other healthcare settings. In addition, this study’s main limitation is that the position and shape of the kidney in the retroperitoneal space may affect the APDRS measurement results. In most people, axial images are approximately vertical to the kidney; however, in rare cases, axial images that are vertical to the kidney cannot be created due to the effect of the organ’s retroperitoneal position and shape; thus, the APDRS measurement value might be affected. In this case, the negative effect on the value measurement can be mitigated by creating vertical axial images of the kidney using multiplanar reformation processing. However, in this study, there were no patients with special kidney positions and shapes as described above.

## Conclusions

We focused on the morphological effects of RSF on the kidneys and found a good relationship between APDRS and RSF and its association with ectopic fat deposition in the abdomen. Furthermore, APDRS expanded in CKD or low eGFR patients than in high eGFR patients. In addition, this measurement method is simple and can be measured on a monitor with measurement tools, contributing to task reduction in the medical field. However, the APDRS does not directly represent the amount of fat deposition because it represents morphological changes in the kidney. Therefore, in the future, the APDRS may provide useful information in evaluating renal diseases by examining its relationship with diseases caused by kidney morphology changes, such as CKD or low eGFR patients.
